# Supplementary material for: Monitoring influenza-like symptoms in the UK through participatory surveillance: Insights from FluSurvey over two winter seasons (2023-24 and 2024-25)
Source: PLOS Digit Health. 2026 Oct 8;5(10):e0001714. doi: 10.1371/journal.pdig.0001714 (PMC13649004; doi:10.1371/journal.pdig.0001714)
Supplement: Supplementary file 1 [file pdig.0001714.s001.docx]

**S1 Table. FluSurvey participant characteristics in the 2023-24 and 2024-25 seasons (England subset only).**
National Census 2021 England data are presented alongside for comparisons.

|  | 2023-2024 | 2024-2025 | Census |
| --- | --- | --- | --- |
|  | *N = 2,237* | *N = 1,999* |  |
| Sex - n (%) |  |  |  |
| Male | 831 (37%) | 707 (35%) | 49% |
| Female | 1,398 (62%) | 1,286 (64%) | 51% |
| Other | 8 (0.4%) | 6 (0.3%) | . |
| Age (years) - mean (SD) | 60 (15) | 60 (15) |  |
| Age group - n (%) |  |  |  |
| 0-17 | 42 (1.9%) | 29 (1.5%) | 20.8% |
| 18-24 | 21 (0.9%) | 18 (0.9%) | 8.3% |
| 25-34 | 57 (2.5%) | 55 (2.8%) | 13.6% |
| 35-44 | 197 (8.8%) | 189 (9.5%) | 13.1% |
| 45-54 | 332 (15%) | 316 (16%) | 13.3% |
| 55-64 | 577 (26%) | 499 (25%) | 12.5% |
| 65+ | 1,011 (45%) | 893 (45%) | 18.4% |
| Ethnic group - n (%) |  |  |  |
| Asian, Asian British or Asian Welsh | . | 28 (1.4%) | 9.6% |
| Black, Black British, Black Welsh, Caribbean or African | . | 10 (0.5%) | 4.2% |
| Mixed or Multiple ethnic groups | . | 24 (1.2%) | 3% |
| Other ethnic group | . | 3 (0.2%) | 2.2% |
| White | . | 1,872 (97%) (97%) | 81% |
| *Missing* | 2,237 | 62 |  |
| Highest level of education- n (%) |  |  |  |
| GCSE / A-level or equivalent | 579 (26%) | 1,414 (71%) | 48% |
| Bachelors degree or higher | 1,562 (70%) | 522 (26%) | 33.9% |
| No formal education | 66 (3.0%) | 42 (2.1%) | 18.1 |
| Still in education | 30 (1.3%) | 21 (1.1%) | . |
| Work activity - n (%) |  |  |  |
| Retired | 990 (44%) | 877 (44%) | 21.5% |
| Full or part-time employment | 932 (42%) | 847 (42%) | 47.7% |
| Self-employment | 116 (5.2%) | 95 (4.8%) | 9.7% |
| Long-term sick or parental leave | 40 (1.8%) | 45 (2.3%) | 4.1% |
| Homemaker | 48 (2.1%) | 57 (2.9%) | 4.8% |
| Unemployed | 16 (0.7%) | 12 (0.6%) | 3.5% |
| Other | 33 (1.5%) | 23 (1.2%) | 3.1% |
| Daycare or education | 62 (2.8%) | 43 (2.2%) | . |
| Smoking - n (%) |  |  |  |
| No | 2,111 (94%) | 1,889 (95%) | . |
| Yes | 97 (4.3%) | 73 (3.7%) | . |
| E-cigarettes | 29 (1.3%) | 36 (1.8%) | . |
| *Missing* |  | 1 |  |
| Allergy - n (%) |  |  |  |
| No | 1,275 (57%) | 1,135 (57%) | . |
| Yes | 962 (43%) | 864 (43%) | . |
| Asthma medication - n (%) |  |  |  |
| No | 1,961 (88%) | 1,751 (88%) | . |
| Yes | 276 (12%) | 248 (12%) | . |
| Diabetes medication - n (%) |  |  |  |
| No | 2,125 (95%) | 1,899 (95%) | . |
| Yes | 112 (5.0%) | 100 (5.0%) | . |
| Lung disease medication - n (%) |  |  |  |
| No | 2,190 (98%) | 1,960 (98%) | . |
| Yes | 47 (2.1%) | 39 (2.0%) | . |
| Heart disease medication - n (%) |  |  |  |
| No | 2,031 (91%) | 1,811 (91%) | . |
| Yes | 206 (9.2%) | 188 (9.4%) | . |
| Kidney disease medication - n (%) |  |  |  |
| No | 2,227 (100%) | 1,987 (99%) | . |
| Yes | 10 (0.4%) | 12 (0.6%) | . |
| Immunocompromised medication - n (%) |  |  |  |
| No | 2,159 (97%) | 1,925 (96%) | . |
| Yes | 78 (3.5%) | 74 (3.7%) | . |
| Influenza vaccination - n (%) |  |  |  |
| Yes, I had one | 1,417 (63%) | 1,433 (72%) | . |
| Yes, I'm planning to | 446 (20%) | 199 (10.0%) | . |
| No | 247 (11%) | 275 (14%) | . |
| I don't know | 127 (5.7%) | 92 (4.6%) | . |
| COVID-19 vaccination - n (%) |  |  |  |
| Yes, I had one | 1,140 (51%) | 1,125 (56%) | . |
| Yes, I'm planning to | 313 (14%) | 120 (6.0%) | . |
| No | 563 (25%) | 689 (35%) | . |
| I don't know | 210 (9.4%) | 59 (3.0%) | . |
| *Missing* | 11 | 6 |  |
| RSV vaccination - n (%) |  |  |  |
| Yes, I had one | . | 146 (7.4%) | . |
| Yes, I'm planning to | . | 58 (3.0%) | . |
| No, I'm eligible but not planning to | . | 50 (2.6%) | . |
| No, I'm not in one of the eligible groups | . | 1,628 (83%) | . |
| I don't know | . | 78 (4.0%) | . |
| *Missing* |  | 39 |  |
