# Supplementary material for: Monitoring influenza-like symptoms in the UK through participatory surveillance: Insights from FluSurvey over two winter seasons (2023-24 and 2024-25)
Source: PLOS Digit Health. 2026 Oct 8;5(10):e0001714. doi: 10.1371/journal.pdig.0001714 (PMC13649004; doi:10.1371/journal.pdig.0001714)
Supplement: Supplementary file 2 [file pdig.0001714.s002.docx]

**S2 Table. Characteristics of influenza-like illness episodes by symptoms, healthcare use, medication, testing, and impact on daily life among FluSurvey participants.**

|  | Overall | 2023-2024 | 2024-2025 |
| --- | --- | --- | --- |
|  | *N = 1,868^1^* | *N = 1,065^1^* | *N = 803^1^* |
| Symptoms - n(%) | | | |
| Cough | 1,477 (79%) | 847 (80%) | 630 (78%) |
| Malaise | 1,384 (74%) | 778 (73%) | 606 (75%) |
| Runny nose | 1,353 (72%) | 794 (75%) | 559 (70%) |
| Sore throat | 1,231 (66%) | 725 (68%) | 506 (63%) |
| Sneezing | 1,169 (63%) | 693 (65%) | 476 (59%) |
| Headache | 1,144 (61%) | 675 (63%) | 469 (58%) |
| Muscle or joint pain | 827 (44%) | 480 (45%) | 347 (43%) |
| Chills | 655 (35%) | 375 (35%) | 280 (35%) |
| Shortness of breath | 600 (32%) | 334 (31%) | 266 (33%) |
| Fever | 601 (32%) | 339 (32%) | 262 (33%) |
| Loss of appetite | 542 (29%) | 285 (27%) | 257 (32%) |
| Coloured sputum | 451 (24%) | 238 (22%) | 213 (27%) |
| Nausea | 251 (13%) | 130 (12%) | 121 (15%) |
| Chest pain | 232 (12%) | 118 (11%) | 114 (14%) |
| Watery or bloodshot eyes | 238 (13%) | 128 (12%) | 110 (14%) |
| Sticky or itchy eyes | . | . | 105 (13%) |
| Loss of taste | 226 (12%) | 121 (11%) | 105 (13%) |
| Loss of smell | 208 (11%) | 124 (12%) | 84 (10%) |
| Stomach ache | 159 (8.5%) | 89 (8.4%) | 70 (8.7%) |
| Diarrhoea | 108 (5.8%) | 53 (5.0%) | 55 (6.8%) |
| Vomiting | 66 (3.5%) | 34 (3.2%) | 32 (4.0%) |
| Nose bleed | 56 (3.0%) | 26 (2.4%) | 30 (3.7%) |
| Any by category - n(%) |  |  |  |
| Gastrointestinal symptoms | 393 (21%) | 211 (20%) | 182 (23%) |
| Ocular symptom | . | . | 182 (23%) |
| Loss of taste or smell | 282 (15%) | 159 (15%) | 123 (15%) |
| Healthcare - n(%) | | | |
| Any healthcare use | 261 (14%) | 136 (13%) | 125 (16%) |
| GP visit | 145 (7.8%) | 68 (6.4%) | 77 (9.6%) |
| GP telephone | 127 (6.8%) | 71 (6.7%) | 56 (7.0%) |
| NHS 111 or online services | 73 (3.9%) | 32 (3.0%) | 40 (5.0%) |
| Hospital admission | 14 (0.7%) | 6 (0.6%) | 8 (1.0%) |
| A&E or out of hours | 42 (2.2%) | 22 (2.1%) | 20 (2.5%) |
| Visited or phoned other  services | 51 (2.7%) | 21 (2.0%) | 30 (3.7%) |
| Medication - n(%) | | | |
| Any medication | 1,665 (89%) | 946 (89%) | 719 (90%) |
| Painkillers | 1,563 (84%) | 893 (84%) | 670 (83%) |
| Cough medication | 409 (22%) | 206 (19%) | 203 (25%) |
| Other medication | 187 (10%) | 103 (9.7%) | 84 (10%) |
| Antibiotics | 147 (7.9%) | 68 (6.4%) | 79 (9.8%) |
| Alternative medicine | 91 (4.9%) | 42 (3.9%) | 49 (6.1%) |
| Influenza antivirals | 6 (0.3%) | 2 (0.2%) | 4 (0.5%) |
| COVID-19 medication | 6 (0.3%) | 6 (0.6%) | 0 (0%) |
| Testing - n(%) |  |  |  |
| Tested for SARS-CoV-2 | . | 511 (48%) | . |
| Tested for influenza | . | 23 (2.2%) | . |
| Tested | . | . | 183 (23%) |
| *Negative result** | . | . | 141 (77%) |
| *Positive result** | . | . | 42 (23%) |
| *- Positive for COVID-19,*  *influenza or RSV** | . | . | 32 (18%) |
| *- Positive for another*  *Infection** | . | . | 10 (5.5%) |
| Impact on daily life - n(%) |  |  |  |
| Daily routine n(%) | . | . | 605 (75%) |
| Missed work n(%)** | 467 (47%) | 270 (46%) | 197 (49%) |
| Minimum healthscore  median [IQR] | 60 [42, 75] | 60 [44, 75] | 59 [40, 75] |
| % difference with no symptoms median [IQR] | 29 [15, 47] | 29 [15, 47] | 29 [14, 48] |

*Proportion testing (N=183) used as the denominator
**Proportion in employment or education used as the denominator (N_2023-24_=584; N_2024-25_=401)
