# Supplementary material for: Monitoring influenza-like symptoms in the UK through participatory surveillance: Insights from FluSurvey over two winter seasons (2023-24 and 2024-25)
Source: PLOS Digit Health. 2026 Oct 8;5(10):e0001714. doi: 10.1371/journal.pdig.0001714 (PMC13649004; doi:10.1371/journal.pdig.0001714)
Supplement: Supplementary file 3 [file pdig.0001714.s003.docx]

**S1 Fig. Bar chart displaying the distribution of participants and new FluSurvey participants by week in each season.** Bars are coloured by reporting status in that week (first report, subsequent report, previously registered and reported that season but did not participate). Both the number (left) and proportion (right) represented are plotted.
