# Supplementary material for: Monitoring influenza-like symptoms in the UK through participatory surveillance: Insights from FluSurvey over two winter seasons (2023-24 and 2024-25)
Source: PLOS Digit Health. 2026 Oct 8;5(10):e0001714. doi: 10.1371/journal.pdig.0001714 (PMC13649004; doi:10.1371/journal.pdig.0001714)
Supplement: Supplementary file 4 [file pdig.0001714.s004.docx]

**S2 Fig. Rates per 1000 FluSurvey participants reporting any symptom, individual symptoms, and meeting the influenza-like illness (ILI) and acute respiratory infection (ARI) case-definition in the 2023-24 and 2024-25 season.** Data are presented for week 47 to week 14, where FluSurvey was operational in both seasons.
