# Supplementary material for: Monitoring influenza-like symptoms in the UK through participatory surveillance: Insights from FluSurvey over two winter seasons (2023-24 and 2024-25)
Source: PLOS Digit Health. 2026 Oct 8;5(10):e0001714. doi: 10.1371/journal.pdig.0001714 (PMC13649004; doi:10.1371/journal.pdig.0001714)
Supplement: Supplementary file 5 [file pdig.0001714.s005.docx]

 **S3 Fig. FluSurvey influenza-like illness (ILI) rates (crude, crude with first survey omitted, age/sex weighted rates) plotted alongside surveillance of B) GP ILI consultations, C) influenza hospital admissions, D) influenza test positivity.** Peak weeks in each system are annotated with a dotted line. Data are presented by week commencing date; as test positivity represented tests conducted the 7 days prior, data from each Sunday were included and shifted back 6 days for consistency with the other systems.
