# Supplementary material for: Monitoring influenza-like symptoms in the UK through participatory surveillance: Insights from FluSurvey over two winter seasons (2023-24 and 2024-25)
Source: PLOS Digit Health. 2026 Oct 8;5(10):e0001714. doi: 10.1371/journal.pdig.0001714 (PMC13649004; doi:10.1371/journal.pdig.0001714)
Supplement: Supplementary file 6 [file pdig.0001714.s006.docx]

**S1 Text. Survey phrasing**

Survey phrasing for the variables derived in the present analyses are included below.

| **Variable** | **Question 2023-24** | **Question 2024-25** | **Notes** |
| --- | --- | --- | --- |
| Country | **What is the first part of your home postcode (the part before the space)?**   - Free text - Don’t know | | *Transformations:*  Postcode mapped to country |
| Sex | **What is your sex?**   - Male - Female - Other | | - |
| Age | **What is your date of birth (year and month)?**  Dropdown month selection Dropdown year selection | | *Transformations:*  Age calculated from month & year of birth |
| Ethnic group | Not asked | What is your ethnic group?  ONS Census 20b [Ethnic group classifications: Census 2021 - Office for National Statistics](https://www.ons.gov.uk/census/census2021dictionary/variablesbytopic/ethnicgroupnationalidentitylanguageandreligionvariablescensus2021/ethnicgroup/classifications) | *Transformations:*  Remapped to ONS Census 6b [Ethnic group classifications: Census 2021 - Office for National Statistics](https://www.ons.gov.uk/census/census2021dictionary/variablesbytopic/ethnicgroupnationalidentitylanguageandreligionvariablescensus2021/ethnicgroup/classifications) |
| Highest education | **What is the highest level of formal education/qualification that you have?**   - I have no formal qualification - GCSE's, O'levels, CSEs or equivalent - A-levels or equivalent (e.g. Higher, NVQ Level3, BTEC) - Bachelors Degree (BA, BSc) or equivalent - Higher Degree or equivalent (e.g. Masters Degree, PGCE, PhD, Medical Doctorate, Advanced Professional Award) - I am still in education | | *Transformations:* GCSE and A-level options combined  Degree options combined |
| Work activity | **What is your work pattern/activity?**   - Paid employment, full time, with at least some work away from home - Paid employment, part time, with at least some work away from home - Working from home (full time) - Working from home (part time) - Self-employed (business owner, farmer, tradesperson, etc.) - Attending daycare/school/college/university - Homemaker (stay at home partner or person with caring responsibilities) - Unemployed - Long-term sick-leave or parental leave - Retired - Other | | *Transformations*: Paid employment and working from home options combined |
| Smoking | **Do you smoke tobacco?**   - No - Yes, occasionally - Yes, daily, fewer than 10 times a day - Yes, daily, 10 or more times a day - I use e-cigarettes only - Dont know/would rather not answer | | *Transformations:*  All “Yes” options combined into “Yes” smoker |
| Allergy | **Do you have one of the following allergies that can cause respiratory symptoms?**   - Hay fever - Allergy against house dust mites - Allergy against domestic animals or pets - Other allergies that cause respiratory symptoms (e.g. sneezing, runny eyes) - I do not have an allergy that causes respiratory symptoms | | *Transformations:* Converted into dichotomous yes/no allergy variable |
| Co-morbidities requiring medication | **Do you take regular medication for any of the following medical conditions?**   - No - Asthma - Diabetes - Chronic lung disorder besides asthma e.g. COPD, emphysema, or other disorders that affect your breathing - Heart disorder - Kidney disorder - An immunocompromising condition (e.g. splenectomy, organ transplant, acquired immune deficiency, cancer treatment) - I would rather not answer | | *Transformations:*  Each response option converted into dichotomous variables |
| Influenza vaccination | **Are you planning to receive a flu vaccine this autumn/winter season?**   - Yes, I'm planning to - Yes, I have got one - No - I don't know | | - |
| COVID-19 vaccination | **Are you planning to receive a COVID-19 vaccine this season?**   - Yes, I'm planning to - Yes, I've had one - No - I don't know | **Are you planning to receive a COVID-19 vaccine this season?**   - Yes, I'm planning to - Yes, I've had one - No, I’m eligible but not planning to - No, I’m not in one of the eligible groups - I don't know | *Changes:* Update to “No” response option in 2024-25  *Transformations:*  “No” options combined in 2024-25 for comparability across seasons |
| RSV vaccination | Not asked | **Are you planning to receive an RSV vaccine? (pregnant women and those aged 75-79 are eligible)**   - Yes, I’m planning to - Yes, I’ve had one - No, I’m eligible but not planning to - No, I’m not in one of the eligible groups - I don’t know | - |

| **Variable** | **Question 2023-24** | **Question 2024-25** | **Notes** |
| --- | --- | --- | --- |
| Symptoms | **Have you had any of the following symptoms since your last visit (or in the past weeks, if this is your first visit)?**   - No symptoms - Fever - Chills - Runny or blocked nose - Sneezing - Sore throat - Cough - Shortness of breath - Headache - Muscle/joint pain - Chest pain - Feeling tired or exhausted (malaise) - Loss of appetite - Coloured sputum/phlegm - Watery or bloodshot eyes - Nausea - Vomiting - Diarrhoea (at least three times a day) - Stomach ache - Loss of smell - Loss of taste - Nose bleed - Other | **Have you had any of the following symptoms since your last visit (or in the past weeks, if this is your first visit)?**   - No symptoms - Fever - Chills - Runny or blocked nose - Sneezing - Sore throat - Cough - Shortness of breath - Headache - Muscle/joint pain - Chest pain - Feeling tired or exhausted (malaise) - Loss of appetite - Coloured sputum/phlegm - Watery or bloodshot eyes - Sticky or itchy eyes - Nausea - Vomiting - Diarrhoea (at least three times a day) - Stomach ache - Loss of smell - Loss of taste - Nose bleed - Other | *Changes:*  Sticky or itchy eyes added in 2024-25  *Transformations:*  Each response converted into dichotomous variables  Reporting any of “vomiting”, “diarrhoea”, “stomach ache”, “nausea” combined into “any GI symptom”  Reporting any of “sticky or itchy eyes” or “watery or bloodshot eyes” converted into “any ocular symptom”  Reporting any “loss of smell” or “loss of taste” converted into “any loss of taste or smell” |
| Sudden onset* | **Did your symptoms develop suddenly over a few hours?**   - Yes - No - I don't know/can't remember | | - |
| Health service use* | **Because of your symptoms, did you VISIT (see face to face) any medical services?**   - No - GP or GP practice nurse - Hospital accident & emergency department / out of hours service - Hospital admission - Other medical services - No, but I have an appointment scheduled | | *Changes:*  NHS Choices separated as response option in 2024-25  *Transformations:*  Telephoning or visiting other medical services combined  GP telephone options combined (receptionist/ doctor)  NHS111/24 and NHS Choices combined in 2024-25 for comparability across seasons  Each response converted into dichotomous variables (e.g. yes/no GP visit) |
|  | **Because of your symptoms, did you contact via TELEPHONE or INTERNET any medical services?**   - No - GP - spoke to receptionist only - GP - Spoke to doctor or nurse - NHS 111 / NHS 24 / NHS Choices - Other - No, but I have an appointment scheduled | **Because of your symptoms, did you contact via TELEPHONE or INTERNET any medical services?**   - No - GP - spoke to receptionist only - GP - Spoke to doctor or nurse - NHS 111 / NHS 24 - NHS Choices - Other - No, but I have an appointment scheduled |  |
| Time to health service use (visit)** | **How soon after your symptoms appeared did you first VISIT a medical service?**  Select the correct number of days:   - Same day - 1 day - 2 days - 3 days - 4 days - 5 days - 6 days - 7 days - 8 days - 9 days - 10 days - 11 days - 12 days - 13 days - 14 days - More than 14 days   I don't know/can't remember | |  |
| Time to health service use (telephone/ internet)*** | **How soon after your symptoms appeared did you first contact a medical service via TELEPHONE or INTERNET?**  **Select the correct number of days:**   - Same day - 1 day - 2 days - 3 days - 4 days - 5 days - 6 days - 7 days - 8 days - 9 days - 10 days - 11 days - 12 days - 13 days - 14 days - More than 14 days - I don't know/can't remember | |  |
| Testing* | **Because of your symptoms, did you get tested for Flu?**   - Yes, swab test - Yes, another test - Not yet but plan to / have an appointment - No - Don't know   **Because of your symptoms, did you get tested for COVID-19?**   - Yes, home swab test (Lateral flow device) - Yes, swab test in healthcare - Yes, another test - Not yet but plan to / have an appointment - No - Don't know | **Because of your symptoms, did you use testing at home or were you tested in any health services in relation to these symptoms?**   - No, I have not been tested - Yes, I tested positive for COVID-19 - Yes, I tested positive for Flu - Yes, I tested positive for RSV - Yes, I tested positive for more than one of COVID-19, Flu or RSV - Yes, I tested positive for another infection - Yes, I was tested but the result was negative - Don’t know/can’t remember | *Changes:* Question and responses updated in 2024-25  *Transformations:* In 2023-24, “Yes” responses combined and converted into yes/no dichotomous variable for both flu and COVID-19  In 2024-25, “Yes” options combined to derive never/ever testing  In 2024-25 “Yes, I tested positive” responses combined into positive result |
| Medication* | **Did you take medication for these symptoms?**   - No medication - Pain killers (e.g. paracetamol including Lemsip and Calpol, ibuprofen, aspirin, etc) - Cough medication (e.g. expectorants) - Influenza antivirals (e.g. oseltamivir/Tamiflu, zanamivir/Relenza) - COVID-19 medical treatments such as Paxlovid, remdesivir or sotrovimab - Antibiotics - Homeopathy - Alternative medicine (essential oil, phytotherapy, etc.) - Other - I don't know/can't remember | **Did you take medication for these symptoms?**   - No medication - Pain killers (e.g. paracetamol including Lemsip and Calpol, ibuprofen, aspirin, etc) - Cough medication (e.g. expectorants) - Influenza antivirals (e.g. oseltamivir/Tamiflu, zanamivir/Relenza) - COVID-19 medical treatments such as Paxlovid or sotrovimab - Antibiotics - Homeopathy - Other alternative medicine (essential oil, physiotherapy, etc.) - Other - I don't know/can't remember | *Changes:* Minor changes to phrasing in 2024-25 for COVID-19 treatments and alternative medicine  *Transformations:*  Homeopathy and alternative medicine combined  Each response converted into dichotomous variables |
| Health score | **On a scale from 0 (bad) to 100 (perfect), how was your health last week?**  Response selected from 0-100 | |  |
| Daily routine* | **Did you change your daily routine because of your illness?**   - No - Yes, but I did not take time off work/school - Yes, I took time off work/school - Not applicable, I don't have work/school | **Did you change your daily routine because of your illness?**   - No - Yes | *Changes:* Question split into two in 2024-25  *Transformations:* “Yes, I took time off work/school” in 2023-24 and “Did you take time off work/school because of your illness” = “Yes” in 2024-25 defined as missing work/school |
| Missing work or school**** | - | **Did you take time off work/school because of your illness?**   - No - Yes - Not applicable (I am not employed or in education) |  |

**Conditional on symptoms selected; **Conditional on visiting healthcare; ***Conditional on telephoning healthcare; ****Conditional on indicating change in daily routine*
